# Reducing Hemoglobin A1C Levels in Type II Diabetes: A Retrospective Analysis of the Renew Procedure

**DOI:** 10.7759/cureus.48039

**Published:** 2023-10-31

**Authors:** Austin F Cross, William Balanoff, Mitchell Karl, Bernard Schayes

**Affiliations:** 1 Research and Development, Renew Limited Liability Company (LLC), Denver, USA; 2 Cardiology, Florida Atlantic University Charles E. Schmidt College of Medicine, Boca Raton, USA; 3 Medicine, Emblem Health Care, New York City, USA

**Keywords:** types 2 diabetes, inflammation, dental implants, dental, oral health, periodontitis, hemoglobin a1c (hba1c)

## Abstract

Introduction: Periodontal disease and type 2 diabetes are interrelated, with inflammation playing a significant role in the progression of both conditions. Previous research has demonstrated the potential of various treatments, such as diet, exercise, and periodontal therapies, to improve glycemic control in diabetic patients.

Method: This study proposed a novel surgical approach, the Renew Procedure, as a potential solution to enhance glycemic control in type 2 diabetic patients with periodontal issues. The procedure involves the removal of all teeth, the elimination of oral and maxillofacial infections, the placement of dental implants, and the provision of implant-supported and implant-retained removable dentures.

Results: Preliminary findings indicated a significant reduction in HbA1c levels post-surgery, suggesting that this approach may improve overall oral health, reduce inflammation, and consequently lower HbA1c levels.

Conclusion: Further research is necessary to confirm the efficacy of the proposed solution, but these initial results highlight the importance of addressing oral health through comprehensive strategies for diabetic and periodontitis patients.

## Introduction

Periodontal disease is one of the most prevalent inflammatory diseases in humans, affecting 40% of all adults over the age of 30 [1]. Furthermore, diabetes is a major public health problem that affects an estimated 34 million people in the United States alone [2]. Type 2 diabetes is a metabolic disorder that results in hyperglycemia due to the body’s inability to produce or use insulin effectively. High levels of hemoglobin A1c (HbA1c), a measure of the average blood glucose level over the previous three months, are associated with an increased risk of complications such as neuropathy, retinopathy, and cardiovascular disease [3]. The inflammatory marker tumor necrosis factor-alpha (TNF-α) has been found to increase insulin resistance in diabetic patients [4], and other inflammatory markers also exacerbate both obesity and type 2 diabetes [5]. These discoveries shifted diabetes research by focusing treatments more specifically on subduing sources of inflammation [6].

Background

It has been shown elsewhere that connections exist between periodontal health and the HbA1c levels of diabetic patients with periodontal issues [7]. The CDC sets out clear guidelines for diagnosing diabetes based on HbA1c levels. A score of 5.7% and below is considered normal; levels ranging from 5.7% to 6.5% are considered ‘prediabetic’, and HbA1c levels above 6.5% are considered to be diabetic [2,8-10].

A major contingency for many of the methods influencing glycemic control is the need for the given patient to adhere to long-term health practices. If a patient is not regularly taking their medications or supplements, exercising, or following a healthy diet, the HbA1c levels will not show much positive change [11].

Here, we propose mitigating the compliance limitation by introducing the Renew Surgical and Prosthetic Procedure. The Renew Procedure involves removing any remaining teeth, removing all oral and maxillofacial infections, removing periodontally involved bone, placing dental implants, and providing implant-supported and implant-retained removable dentures. Since HbA1c is affected by inflammatory disease, by removing a primary mitigating contributing factor to the inflammatory disease mechanism (periodontal disease), we hoped to see a reduction and stabilization of baseline HbA1c levels in type 2 diabetic patients. We hypothesized that introducing the Renew Procedure to a patient who qualified for it would lead to improved overall oral health by eliminating the chronic inflammation in the patient’s mouth, leading to reduced baseline HbA1c levels. Furthermore, having a complete set of functioning teeth with improved oral health can lead to better diets and improve the overall quality of life, which itself may reduce HbA1c and thus further augment the more direct anti-inflammatory benefit of surgery.

Periodontitis has been linked to elevated HbA1c levels, and there is evidence that proper treatment, paired with overall improvements in oral health, can improve glycemic control in type 2 diabetic patients [12]. Periodontitis is a chronic inflammatory disease caused by bacteria (plaque) that accumulate on the teeth. It leads to immune and inflammatory responses from the body, specifically the supporting tissue of the teeth. Studies have shown that diabetic individuals have both a higher occurrence and severity of periodontitis compared to non-diabetic individuals [13]. The relationship between diabetes and periodontitis is bidirectional, meaning that either can exacerbate the other. There is a pathologic synergy when both conditions are simultaneously present [14]. The mechanism by which periodontitis affects glycemic control is not entirely understood, but evidence suggests that the chronic inflammatory response associated with periodontal disease contributes to insulin resistance and hampered glucose uptake [15]. Additionally, the bacterial components of the plaque can cause immune cells to release inflammatory mediators, which also promote insulin resistance [16]. Periodontitis is typically treated by mechanical debridement of the bacteria (plaque) and other irritants from the teeth and/or gums. This is achieved through scaling and root planning (SRP), a non-surgical procedure where the dental hygienist removes plaque and calculus buildup from the teeth [17]. In some cases, antibiotics can be prescribed systemically and locally. A systematic review by Gaurav found that periodontal treatment, specifically SRP, was associated with a significant reduction in HbA1c levels in diabetic patients, with a mean reduction of 0.4% [18].

Anti-inflammatory antibiotics, such as doxycycline, have the potential to treat periodontitis and manage type 2 diabetes when used in their local form. Doxycycline, when used in tandem with SRP, has beneficial effects on periodontal inflammation [19]. According to the authors, doxycycline with SRP resulted in significant drops in probing pocket depth while also improving clinical attachment levels compared to SRP alone. The direct impact of doxycycline on type 2 diabetes is less established, but there is evidence that its anti-inflammatory properties may have a positive effect on diabetic complications. Doxycycline reduces heart-related complications induced by diabetes by reducing inflammation and oxidative stress [20].

In general, patients with diabetes who can lower their HbA1c levels will also see lowered risks for complications that arise from having diabetes long-term, including retinopathy, kidney failure, or even death [21]. 90% of patients with type 2 diabetes included in a long-term study focused on HbA1c levels exhibited significant variability (≥0.5% change in 1 year) [22]. It was also found that higher HbA1c levels and poor glycemic control led to a greater risk for HbA1c variability. Inversely, in order to achieve a lower risk for HbA1c variability, lowered baseline HbA1c levels and increased glycemic control must be achieved. Moreover, other studies have shown that variability in HbA1c is directly associated with increased risks for emergency hospitalizations and mortality [23]. The authors demonstrated that average HbA1c levels are still important factors in overall health risks, particularly for patients in the higher ranges of HbA1c readings (over 9%). Patients below that 9% threshold were hypothesized to need to focus on the stability of readings over the lowering of average readings. Given that only N=3 of N=44 total patients were above the 9% A1C threshold pre-surgery, it was determined that focusing on lowering averages would be more insightful than tightening the variability.

Given the strong positive association between HbA1c levels and diabetic complications and the association of higher HbA1c levels with periodontal disease, we tested the hypothesis that a surgical procedure that replaces teeth and markedly reduces oral inflammation would reduce HbA1c as a proxy for reducing diabetic complications.

## Materials and methods

Patients were taken from the database of past and current patients at Renew Dental who had received the Renew Procedure prior to the initial query search. As per the Renew operative process, all patients signed informed consent and release of records forms before or after their surgery, permitting their medical data to be used in HIPAA-protected studies to determine the health benefits and patient satisfaction of the Renew procedure. Information was initially queried by keywords, such as 'diabetes' and 'A1c', searching for patients who had received the surgery already, as well as patients with type 2 diabetes mellitus. The study examined the records of patients who met a certain set of eligibility criteria.

First, these patients were at least 18 years of age at the time of the study, with no upper age limit being set. They had received their surgeries at any point prior to the initial query date of September 2022. Additionally, patients had been diagnosed with type 2 diabetes for at least one year at the time of surgery. Finally, for those who were diagnosed less than a year prior to their surgery, the study required that their pre-surgery HbA1c levels be within or exceed the Centers for Disease Control and Prevention's (CDC) defined range for 'pre-diabetes', which is a level of 5.7 or above.

After applying the inclusion criteria, a population of 83 patients with T2D was generated. Medical records were not on file for post-operative healthcare visits and had to be requested through primary care providers or through personal communication with patients (if their signed Release of Records forms were outdated). Some patients agreed to sign and update their records, while others provided their HbA1c information during a phone call. Exclusion criteria included a failure to provide informed consent or follow-up data from post-surgical healthcare visits, resulting in a final sample population of n=44.

These patients were placed into a spreadsheet without personal identification outside of their Renew Patient Numbers. The collection of data encompassed a diverse array of criteria. To begin with, the exact date of the surgery for each patient was recorded, and information on the patient's sex was also noted. Additionally, the patient's age at the time of surgery was registered. The determination of whether a patient was a regular smoker or drinker was made based on their responses to 'Do you smoke or drink?' Yes/No questions are featured on the Renew Health History Intake Form. Acknowledging the impact of these choices on health outcomes, they were variables to be considered within the study.

The study also recorded the pre-surgery HbA1c values, provided they were available within the nine months leading up to the surgery. The exact 'time pre-op' - the duration before the surgery when these values were gathered - was calculated using the DAYS function on Excel (Microsoft® Corp., Redmond, WA). Finally, the study included the collection of post-surgery HbA1c values and the dates of these lab tests. From these, the 'time post-op', denoting the duration after the surgery when the analyses were conducted, was calculated.

Statistical analysis

In an effort to gain a comprehensive understanding of the effects of the surgery on patients' health, a comparative analysis of pre- and post-surgery average HbA1c values was conducted. Initially, the entire sample from the study was considered. This provided an overview of the broad impact of the surgery on HbA1c levels. Subsequently, the data were broken down according to sex, comparing male and female patients. This was crucial to identify any potential sex-specific trends in the effect of surgery on HbA1c levels. Finally, lifestyle factors were taken into account. Patients were divided into groups of smokers, drinkers, those who were both, and those who were neither, and their pre- and post-surgery HbA1c values were compared. This analysis aimed to uncover the potential interactions between lifestyle choices and the effects of the surgery.

The main focus of the research was on the HbA1c trends for the entire sample population, as the overall goal was to understand more about the effect of the Renew Procedure on all patients with type 2 diabetes. The categories of smokers, drinkers, both, and neither were included due to the known connections between these activities and inflammation in the body [24].

## Results

**Figure 1:**
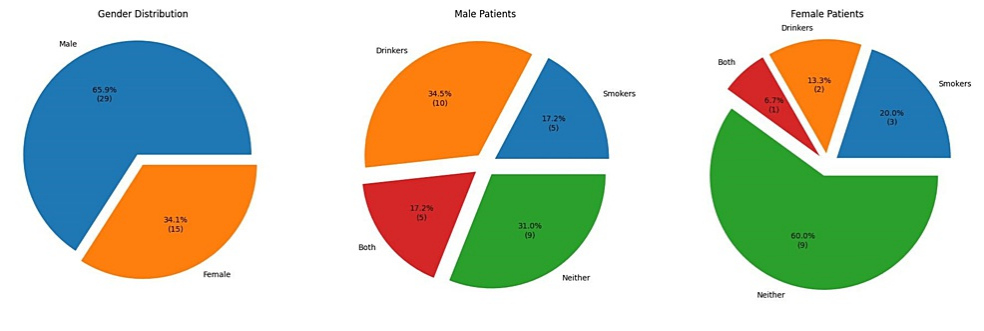
Gender distribution of patients.

**Figure 2:**
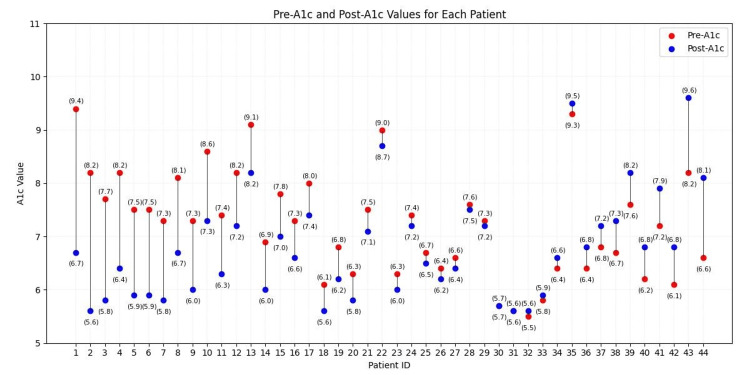
Pre- and post-A1c values for each patient.

Patients ranged in age from 39 to 83 on the day of surgery. Figure 1 provides an overview of the sample by sex (female, male) and practices (smoking, drinking, both, or neither). Initial pre-surgery HbA1c values for the sample ranged from 5.5 to 9.1, with 12 (27.27%) patients’ initial values below the 6.5 mark set by the CDC guidelines for being considered diabetic and instead in the ‘pre-diabetes’ range. Post-surgery HbA1c values ranged from 5.6 to 9.6, with 19 (43.18%) patients at or below 6.5. Figure 2 provides an overview of pre- and post-values by patient.

The average elapsed time for pre-surgery readings was 108.75 days pre-op, while the average time for post-surgery readings was 301.34 days post-op. Table 1 lists the group-level averages for pre- and post-op’ A1c readings. As can be seen, the eight regular smokers saw a 12.50% (0.925 A1C points) average drop in their HbA1c values post-surgery, making this the subgroup with the largest average change. The 12 regular drinkers dropped 3.13% on average (0.217 A1C points). The six patients who identified as regular smokers and drinkers saw their HbA1c values drop an average of 3.67% (0.267 A1C points), and the eighteen patients who identified as neither smokers nor drinkers saw an average HbA1c drop of 6.46% (0.478 points) after surgery. Comparing across groups, there were varying ranges of change trends in directions that we anticipated. Regular smokers had the highest average pre-surgery HbA1c values (7.40), and regular drinkers had the lowest (6.925). The average post-surgery HbA1c values ranged from 6.475 (smokers) to 7.00 (both smokers and drinkers). The smaller range of post-surgery HbA1c values potentially indicated a loosely defined trend of stabilization among HbA1c values after surgery compared to the values before surgery. Research has shown that stabilizing the variance of HbA1c values can be more beneficial to positive outcomes in long-term health than simply lowering HbA1c average values [25].

**Table 1:** Group and subgroup counts, average, standard deviations, and pre- and post-differences.

Group	(N)	Pre Avg	Pre SDv	Post Avg	Post SDv	Avg Diff
All	44	7.250	7.009	6.791	1.006	−0.459
Men	29	7.086	1.072	6.672	1.027	−0.414
Women	15	7.567	0.912	7.020	0.925	−0.547
Smoke	8	7.400	1.363	6.475	0.886	−0.925
Drink	12	6.925	1.125	6.708	1.089	−0.217
Both	6	7.267	0.585	7.000	1.032	−0.267
None	18	7.394	0.885	6.917	1.034	−0.478

To test the hypothesis that there will be a significant decrease in HbA1c values from pre-surgery to post-surgery, a paired sample t-test was conducted. There were no outliers in the data, as assessed by an inspection of a boxplot. The differences between the pre-test and post-test of HbA1c were normally distributed, as assessed by Shapiro-Wilk's test (p = 0.739). Patients’ HbA1c results were lower post-surgery (mean [M] = 6.791, standard deviation [SD] = 1.006) than pre-surgery (M = 7.250, SD = 1.009). Specifically, there was a mean decrease in HbA1c scores of −0.4591, 95% CI [−0.7463, −0.1719] from pre- to post-surgery. The paired-sample t-test revealed the decrease in HbA1c was statistically significant [t(43) = −3.224, p < 0.001]. In addition, results revealed a medium effect size (d = −0.486), suggesting that on average, the A1c results post-surgery were almost 0.5 standard deviations lower than pre-surgery HbA1c results.

The null hypothesis was that there would be no significant difference between pre-surgery and post-surgery HbA1c values or that the mean of the differences in HbA1c values was zero. Results revealed support for the alternative hypothesis that a significant difference between pre-surgery and post-surgery HbA1c values would occur in the study population. The calculated t-value (−3.224) had an absolute value greater than the critical t-value. This provides evidence to reject the null hypothesis, as there was a significant difference between pre-surgery and post-surgery HbA1c values.

To gain a better understanding of the 13 patients who exhibited higher HbA1c values post-surgery compared to pre-surgery values (two patients exhibited zero change in A1C values from pre-to-post), five patients were contacted by phone. Of those five, four reported a secondary diagnosis or chronic condition that was preventing them from either staying on their medications, eating healthily post-op, or properly recovering from the operation (two cancer diagnoses/undergoing chemo currently, one IBS diagnosis causing health and diet issues, and one very volatile case of type 2 diabetes that the patient and their doctors were still working on controlling). The fifth patient stated over the phone that they stopped taking all diabetes-related medications in the months leading up to their surgery and had not begun taking them again. They stated that they felt better off the medications, even though it caused their HbA1c levels to be higher. This specific patient saw their HbA1c levels go from 6.8 a month before the surgery to 7.2 about 15 months later. This highlights that receiving the surgery alone is not enough; patients must adhere to the recovery process as outlined by the Renew surgical procedure.

## Discussion

The present study underscores the relationship between gingival inflammatory disease and hemoglobin A1C levels in diabetic patients. It further demonstrated that a surgical intervention aimed at improving both patient aesthetics and functionality is able to reduce HbA1c. As HbA1c levels are a proxy for multiple diabetic complications, the likely health benefit derived from this surgery could be significant.

Preliminary findings indicated a potential relationship between positive overall health behaviors, such as diet and exercise, and the Renew Procedure for patients with type 2 diabetes. Patients on average saw a drop in HbA1c values within the first year post-surgery at Renew, due to eliminating a major source of inflammation in their bodies, which is a well-documented stressor for T2DM that leads to elevated HbA1c values [6]. Given access to a wider source of data over time, we expect to see similar trends continue with our research. Furthermore, if the patient was also a regular smoker, eliminating this from daily activities lowered the HbA1c values even further. This is shown in the drop in HbA1c levels of smokers after surgery, as part of the recovery process involves abstaining from smoking while the oral surgical scars and incisions heal.

Elevated HbA1c levels in diabetics are linked to respiratory elements, retinopathy, neuropathy, nephropathy, and cardiovascular mortality [26]. A surgical procedure that reduces HbA1c could represent an adjunct or alternative to medications that may otherwise have significant side effects. Although the focus of the present study was the change in HbA1c related to surgical intervention, this is only one potential benefit of the Renew surgery.

Poor dentition is also associated with gingival inflammation. This is best thought of as a systemic process that may lead to arterial inflammation and plaque instability. The latter can lead to heart attacks, strokes, and sudden cardiac death. Researchers have confirmed endothelial dysfunction, abnormal arterial stiffness, increased carotid plaque burden, and calcium scoring levels in patients with periodontal disease [27,28]. Thus, periodontal disease is associated with increased atherosclerosis and a greater likelihood of plaque instability from inflammation, which in turn leads to cardiovascular events. Because the Renew procedure addresses the substrate for oral inflammation, it would be expected to reduce plaque formation and plaque vulnerability while materially improving cardiovascular outcomes. Further studies are needed to determine to what extent the Renew procedure can offer this cardiovascular benefit.

Diabetes and poor oral health are interconnected, but oral health can have a significant impact on systemic health as well [29]. A meta-analysis reviewed the evidence linking poor oral health to a range of other conditions, including diabetes, respiratory infections, and cardiovascular disease [29]. The author emphasized the importance of maintaining proper oral hygiene and seeking prompt treatment for dental conditions as a promotion of overall health and well-being.

In order to confidently hypothesize on the connections between periodontitis, type 2 diabetes, and the Renew Procedure, it is necessary to acknowledge the potential shortcomings and confounders present in this retrospective study so as to properly direct the focus of future research on the topic. First, the lack of well-defined inflammatory markers being tested in the patient records data leads to more assertion than conclusion, and an expanded dataset with more specific tests for inflammatory markers can help narrow the focus of our conclusions going forward. As for the potential confounders present, it is possible that the patients were greatly influenced by the surgical recovery. Patients can be greatly affected by the recovery process, resulting in lowered levels of food intake during recovery, potentially leading to weight loss and lowering inflammation. In order to investigate the true influence that the surgery is having on these markers of inflammation, future studies should aim to look at pre- and post-surgery markers of inflammation in tandem with the HbA1c levels, while also monitoring certain variables such as weight, diet, and exercise changes pre- and post-surgery.

## Conclusions

The growing body of evidence linking periodontitis and diabetes highlights the importance of the development of a comprehensive surgical process aimed at improving multiple aspects of a patient's health. The Renew procedure, which includes dental implants and implant overdentures, has shown promise as an effective treatment option for chronic oral health conditions in diabetic patients. More broadly, periodontitis management is critical to achieving optimal glycemic control and reducing the risk of diabetes-related complications. Preliminary evidence suggests that the Renew surgery can not only restore aesthetics, functionality, and a youthful appearance but also be a significant step toward reducing chronic disease and improving health.
